# Oral cancer in the UAE: a multicenter, retrospective study

**DOI:** 10.3402/ljm.v8i0.21782

**Published:** 2013-08-27

**Authors:** Raeefa Anis, Kamis Gaballah

**Affiliations:** 1 Mafraq Dental Center, Abu Dhabi, UAE; 2 Department of Oral and Maxillofacial Surgery, College of Dentistry, Ajman University, Ajman, UAE

**Keywords:** UAE, oral cancer, biopsy, prevalence, early detection, neck dissection

## Abstract

**Aim:**

To determine the prevalence of various malignant oral lesions in the UAE and correlate cases of squamous cell carcinomas with age, gender, site, grade, clinical presentations at the time of diagnosis, and the prevalence of neck metastasis.

**Materials and methods:**

A multicenter, retrospective study was conducted at four major hospitals in the UAE. The study was based on histopathology reports of biopsies of oral tissues.

**Results:**

Of the 992 oral biopsy reports retrieved, 147 cases of malignant tumors were found which accounted for 14.9% of the total biopsies. Fifteen different types of malignant lesions were diagnosed, of which oral squamous cell carcinoma (OSCC) was the most prevalent and made up 11.4% of the overall oral biopsies retrieved. The commonest presentation of cancer was ulceration (31.17%), followed by lumps and white lesions. The most common site where the lesions were diagnosed was the tongue (51.9%), followed by the cheeks and lips. OSCC accounted for 77% of all malignancies reported. Neck dissections were conducted in only 20.8% of all OSCC cases diagnosed at Mafraq and Tawam hospitals, of which 43.75% showed evidence of neck metastasis.

**Conclusion:**

Oral cancer is not an uncommon disease in the UAE. This may mandate more awareness campaigning, including screening procedures for early detection of cancerous lesions and other potentially malignant oral diseases. Elective neck dissections to detect lymph node metastasis should be more routinely performed, in particular for tongue carcinomas because of the early neck involvement potential.

Oral cancer, with an annual incidence of over 300,000 cases, is reported to be the eighth most common cancer globally (1, 2). Incidence and mortality as a result of oral cancer are higher in developing countries when compared to developed countries (2, 3). According to the latest World Health Organization (WHO) data recorded in 2010, the death rate due to oral cancer in the Middle East is reported to be approximately 2 in 100,000, which is much lower than that in India and in the United States (4).

Squamous cell carcinomas (SCCs) of the lip and oral cavity comprise 90–95% of all oral malignancies. Hidden regional metastasis in oral squamous cell carcinoma (OSCC) is prevalent in at least 30% of cases (5). Clinical examination alone is proven to be unreliable in detecting such regional metastasis. Identification of regional metastasis and early intervention could decrease mortality rates. Improved diagnostic modalities are required not only to detect regional disease but also to decrease post-operative morbidity and mortality.

This study aims to determine the prevalence of various malignant oral lesions in the UAE, to correlate cases of OSCC with age, gender, site, grade, clinical presentation, and neck metastasis identified.

## Materials and methods

A multicenter, retrospective study of oral biopsies was conducted in four hospitals in the UAE. Oral biopsy reports were retrieved from Tawam Hospital in Al Ain and Al Mafraq Hospital in Abu Dhabi. Data were taken from Tawam Hospital and Al Mafraq Hospital, and data from the Iranian and Al Baraha Hospitals in Dubai were also reviewed (6, 7). Data recorded included age, sex, site of the lesion, clinical presentation, histological grade, and information pertaining to neck dissections, if any. The distribution of the cases is shown in Table 1. A more detailed analysis was conducted for cases of OSCC diagnosed at the Mafraq and Tawam Hospitals. Cases of OSCC included all cancers of the oral cavity (i.e. those found on the lips, tongue, buccal mucosa, retromolar region, palate, and other areas of the oral cavity). Cases of tonsillar carcinomas and pharyngeal cancers were not included. Data were subjected to descriptive analysis using Microsoft Excel 2007.

**Table 1:** The distribution of the cases among various hospitals

Hospitals	Study years	Total no. of oral biopsies retrieved	Malignant lesions	OSCC
Tawam	2008–2011	223	74	60
Mafraq	2009–2012	248	21	17
Baraha	2005–2011	133	44	3
Iranian	2007–2010	388	8	23

*Note:* OSCC, oral squamous cell carcinoma.

## Results

**Fig. 1:**
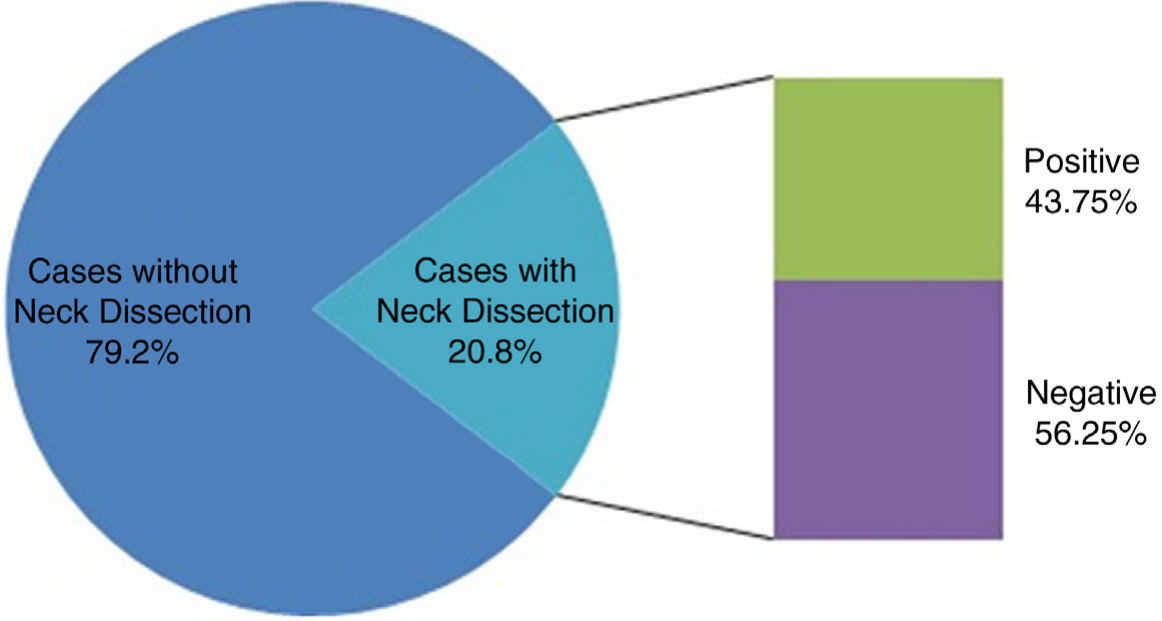
Neck dissection outcome for 77 cases with squamous cell carcinoma.

Of the 992 oral lesions biopsied in the specified time periods at the four hospitals, a total of 147 malignancies pertaining to the oral cavity were identified. Analysis of the records showed that the most prevalent malignant lesion was OSCC, followed by mucoepidermoid carcinoma of the salivary glands (Table 2). A total of 113 cases of OSCC were diagnosed, which makes up 77% of the total malignancies biopsied. Of the 77 cases of OSCC diagnosed at the Tawam and Mafraq Hospitals, 62 were found in males and 15 in females, which corresponds to a male-to-female (M:F) ratio of 4.13. For 77 cases of OSCC, the average age at diagnosis of OSCC was 54.9 years with a standard deviation of 12.99 years and an age range of 28–89 years. The commonest site of diagnosis of OSCC was the tongue, which represents 51.9% of the sample, followed by the buccal mucosa (19.48%) and lip (11.6%). Of the lesions diagnosed as SCCs, 31.17% presented clinically as ulcers, followed by lumps (18.18%) and white lesions (3.9%). Well-differentiated OSCC accounted for two-thirds of the samples examined (62.3%), followed by moderately differentiated (20.8%) and poorly differentiated OSCC (6.5%). Among the 77 cases of SCC, 16 patients (20.8%) had neck dissections performed, of which 43.75% were positive and 56.25% were negative for metastasis (Fig. 1).

**Table 2:** Distribution of the various histopathological diagnosis of malignant lesions in the UAE for the studied time periods

Diagnosis	Frequency	Percentage of malignant tumors (%)
Malignant neoplasms of epithelial origin		
Squamous cell carcinoma	103	70
Papillary carcinoma (variant)	8	5.4
Spindle cell carcinoma (variant)	2	1.4
Malignant melanoma	2	1.4
Total: 115		78.2
Malignant neoplasms of glandular origin		
Mucoepidermoid carcinoma	8	5.4
Adenoid cystic carcinoma	4	2.7
Adenocarcinoma	4	2.7
Malignant salivary gland tumor (type unspecified)	2	1.4
Clear cell carcinoma	1	0.7
Salivary duct carcinoma	1	0.7
Total: 20		13.6
Malignant neoplasms of mesenchymal origin		
Rhabdomyosarcoma	4	2.7
Ewing's sarcoma	2	1.4
B-cell lymphoma	2	1.4
Burkitt's lymphoma	2	1.4
Plasma cell tumor	2	1.4
Total: 12		8.3
Total	147	100

## Discussion

The incidence of malignant neoplasms varies from one country to another, which can be explained by the difference in the distribution of the risk factors and the possible etiologies. In this report, we compare the pattern of oral cancer with that in countries with some similarities with the UAE population. In this regard, the disease appears in 1.8%, 1.4%, 0.4%, 2.5%, and 8% of the oral biopsies reviewed in a Turkish, Spanish, Cambodian, Brazilian, and Libyan population, respectively (8–12). These were much lower than the prevalence of malignant tumors found in our series, which was 14.9% of all oral biopsies. On the contrary, a much higher prevalence of malignant lesions was reported in a similar study in Pakistan (55.8%) (13) and Nigeria (18%) (14). These differences may be attributed to the trend of surgical biopsy of oral lesions; when more lesions are sampled, the percentage of oral cancer reported will be diluted. It is quite clear that many dentists do not practice biopsy of the lesions, so this procedure is mainly carried by surgeons for the referred cases that look suspicious to either the referring dentist or the surgeon. Training students and junior dentists should know how to perform biopsy of oral lesions as it may help to identify many patients with early cancer or even with potentially malignant oral lesions. The most prevalent diagnosis of all oral biopsies was OSCC, which accounted for 77% of all oral malignancies. A much lower proportion was reported by a study conducted in Libya (41%) (12). Conversely, OSCC constituted a higher proportion of oral malignancies in Jordan (84%) (15), Malaysia (91.3%) (16), and Pakistan (92.2%) (13). The M:F ratio for OSCC in our study was found to be 4.13, which is consistent with the findings in Portugal and India (17, 18). This differed slightly from the findings in Brazil and Sri Lanka, where the M:F ratio was 3:1 (11, 19). Lower M:F ratios were seen in Iraq (2:1) (20), Jordan (1.77:1) (15), Libya (1.61:1) (12), Pakistan and Nigeria (1.4:1) (13, 21), Iran and Thailand (1.3:1) (22, 23), Yemen (1.24:1) (24), and Malaysia (0.92:1) (16). Gender is not a risk factor *per se* for oral cancer. Our findings may reflect the fact that an increased consumption of tobacco and alcohol is more common among men than women. An average age of 54.9 years was found with a standard deviation of 12.99 years, similar to the findings in India and Pakistan (55 and 51.9 years) (18, 25). An age range of 28–89 years was noted in our study. Even wider age ranges of 10–80 years swere seen for OSCC in studies conducted in Allahabad, India (18), Yemen (16–110 years) (24), Nigeria (3–86 years) (21), Thailand (17–97 years) (23), and Libya (21–93 years) (12).

In recent years, increasing trends of oral cancer in younger people have generated interest in several regions of the world. The etiology could be early indulgence in tobacco or alcohol consumption. Highest odds ratios for oral cancer were associated with commencement of smoking before the age of 16 years (26). Interestingly, young never-smokers and never-drinkers were diagnosed with OSCC, wherein a link with human papillomavirus has been suggested (26). However, variations exist in the cutoff point defined for young people. In our series, 14.3% of OSCC were below 40 years. Comparable results were reported in Yemen (14%) (24), Iran (13%) (22), and Libya (15%) (12). A lower proportion of younger cases of OSCC were seen in Brazil (8.7%) (11), Sri Lanka (8.7%) (19), and Malaysia (4.3%) (16). In contrast, studies performed in Nigeria and India reported 40% and 17% of young people with OSCC, respectively (21, 27).

Numerous studies defined young people as 45 years and below. Twenty-six percentage of OSCC patients were younger than 45 years in our study, which was comparable to findings in Yemen (24), but higher than those reported in Jordan (15), Brazil (11), Thailand (23), the United States (28), and the United Kingdom (29). The diagnosis of cancer at a younger age indicates the need to biopsy suspicious oral lesions to rule out malignancy in patients as young as in their second or third decade of life.

Of the 77 cases of OSCC in our study, the most common site was the tongue (51.9%), followed by the buccal mucosa (19.4%). The lip, the third common site, formed 11.6% of all cases of SCC. Tongue is also reported to be the most common site, followed by buccal mucosa, in Libya (12), India (18, 30), and the United States (28). Two factors that can explain the high risk involving these sites is that carcinogens mixed with saliva pool at the bottom of the mouth and constantly bathe these sites. Second, these regions of the mouth are lined by a thinner, non-keratinized mucosa and hence provide decreased protection against carcinogens (12). Contradictory findings were reported in Pakistan, wherein buccal mucosa was the most common site followed by the tongue and palate (13, 25). Extensive use of betel quid in this region could explain why buccal mucosa was the most common site in their series. In Nigeria (21), the gingiva is reported to be the most common oral subsite, whereas the lip is the predominant site in Iraq (20).

In this study, the majority of the cases was found to be well-differentiated carcinomas and comprised 62.3% of the sample, followed by moderately differentiated (20.8%) and poorly differentiated carcinomas (6.5%). Well-differentiated carcinomas were also most common in Iraq, Libya, and India (12, 18, 20, 30). Contrarily, moderately differentiated carcinomas formed the majority of OSCC in Pakistan (25), whereas poorly differentiated carcinomas were most common in Nigeria (21). The investigation of Tawam Hospital records in 2010 (31) showed that potentially malignant lesions were detected in 20.7% of oral mucosa biopsies. This figure is quite vital to the chance of early detection of malignant lesions. The identification of potentially malignant changes should initiate a coordinated process of risk assessment and close follow-up by both specialist surgeons as well as general dental practitioners. However, clinical presentations of OSCC at the time of diagnosis were predominantly ulcers, representing 31.7% of the sample, followed by lumps and swellings, which accounted for 18.2% of the cases of SCC. Interestingly, in this series there were three cases (3.9%) of OSCC preceded by leukoplakia, which is consistent with international literature, which report that between 3 and 33% of oral cancer can develop from such lesions (32).

The current report shows that 16 of the 77 cases of SCC (20.8%) included neck dissections. Of the 16 neck dissections, 7 were positive for lymph node metastasis, which made up 43.75% of the neck dissections and 9.1% of the cases of OSCC (Fig. 1). Akhtar et al., in their study, stated that carcinomas of the tongue metastasize more frequently than carcinomas of other regions of the oral cavity (33). The detection of lymph node involvement in the absence of clinical and radiological evidence is defined as occult metastasis or micrometastasis. In approximately 30% of the patients with early carcinoma, occult metastasis has been noted. The same researchers reported in their sample of tongue carcinomas that lymph node metastases for stage T1 carcinomas were positive in 28% of the neck dissections, and T2 carcinomas were positive for 34% of them. An overall rate of 32% for occult lymph node metastases in T1 and T2 carcinomas was hence reported (33).

In our series, 43 out of 77 OSCCs were carcinomas of the tongue, of which only nine underwent neck dissections and represent 20.9% of the tongue carcinomas. Five of these were positive for lymph node metastasis and account for 11.6% of the cases of tongue carcinomas. Although the stage of cancer is not known for our sample, assuming that all carcinomas of the tongue were at stage T1 or T2, the percentage of positive neck dissections, which was 11.6%, is much lower than percentages reported in the literature (32% and 34%) (33, 34). Micrometastasis is thus a possibility in a percentage of the remainder of tongue carcinomas which may have been overlooked. Tongue SCC has a 5-year survival rate of 73% in pN0 cases, regardless of the T stage. Survival rate decreases to 40% in patients with positive nodes without extracapsular spread (pN1 ECS−) and falls to 29% when nodes are metastatic with extracapsular spread (pN1 ECS+) (26). Detection of nodal micrometastasis is thus a key element in evaluating prognosis.

Another study states that nodal micrometastasis is found in up to 50% of cN0 pN1 cases of tongue carcinomas (35). Neck dissection in these cases is highly debated and could be considered an overtreatment in half or more than half of these cases. Consequential morbidity includes hemorrhage, nerve injury, lymphedema, and pain. However, the wait-and-watch policy proposed by some could be considered an undertreatment and could worsen the prognosis of the patient in almost half of cN0 tumors (35).

The most selective form of neck dissection, called ‘sentinel node biopsy’ (SNB), has been recommended, and its efficacy has been proven by a meta-analysis published in 2007 (5). It involves the use of lymphoscintigraphy, vital dye, and gamma probe to detect the sentinel node. The sentinel node is the first drainage node where a solid tumor metastasizes. The concept rests on the fact that if the sentinel node is free of metastasis, more distal nodes are also disease free (5). Although widely used in cases of breast cancer and melanoma, this procedure is yet to be adopted for oral cancer in many countries (5, 26). Studies on the sensitivity and specificity of SNB showed that the technique was more accurate than the other imaging techniques available for detection of neck node involvement (5, 35) (Table 3).

**Table 3:** Comparison of the sensitivity and specificity values of different diagnostic procedures to detect micrometastasis in cN0 patients (5, 35)

Diagnostic procedure	Sensitivity (%)	Specificity (%)
CT	52	93
MRI	65	81
PET	66	87
US	66	78
SNB	93	100

*Note:* CT, computed tomography; MRI, magnetic resonance imaging; PET, positron emission tomography; US, ultrasound; SNB, sentinel node biopsy.

In conclusion, about 15% of oral biopsies revealed the presence of malignant disease. The prevalence of oral cancer lies between that in Western and South-east Asian populations. OSCC typically affected people older than 50 years but with a proportion affecting the younger age group. A considerable number of patients may be undertreated as the neck dissection was performed on only one-fifth of patients. This may be partly due to lack of appropriate staging or lack of surgical expertise to carry such complex surgeries. In this regard, the introduction of SNB is recommended to identify patients who require neck dissection to minimize the locoregional recurrence and improve the survival rate. Screening for oral cancer has also proved successful in reducing the incidence and mortality caused by OSCC. The authors advocate oral cancer screening in the UAE to reduce the incidence of the disease and detect potentially malignant lesions to prevent its transition to frank carcinoma.
